# Supplementary material for: Causal interplay between lactose intolerance and gut microbiota: a combined bidirectional Mendelian randomization and in vivo validation study
Source: Front Nutr. 2026 Jun 1;13:1803337. doi: 10.3389/fnut.2026.1803337 (PMC13265576; doi:10.3389/fnut.2026.1803337)
Supplement: Supplementary file 1 [file Data_Sheet_1.zip › supplementary materials/Forward/leave one out cross validation/ebi-a-GCST90027499.finngen_R12_E4_LACTONAS.pdf]

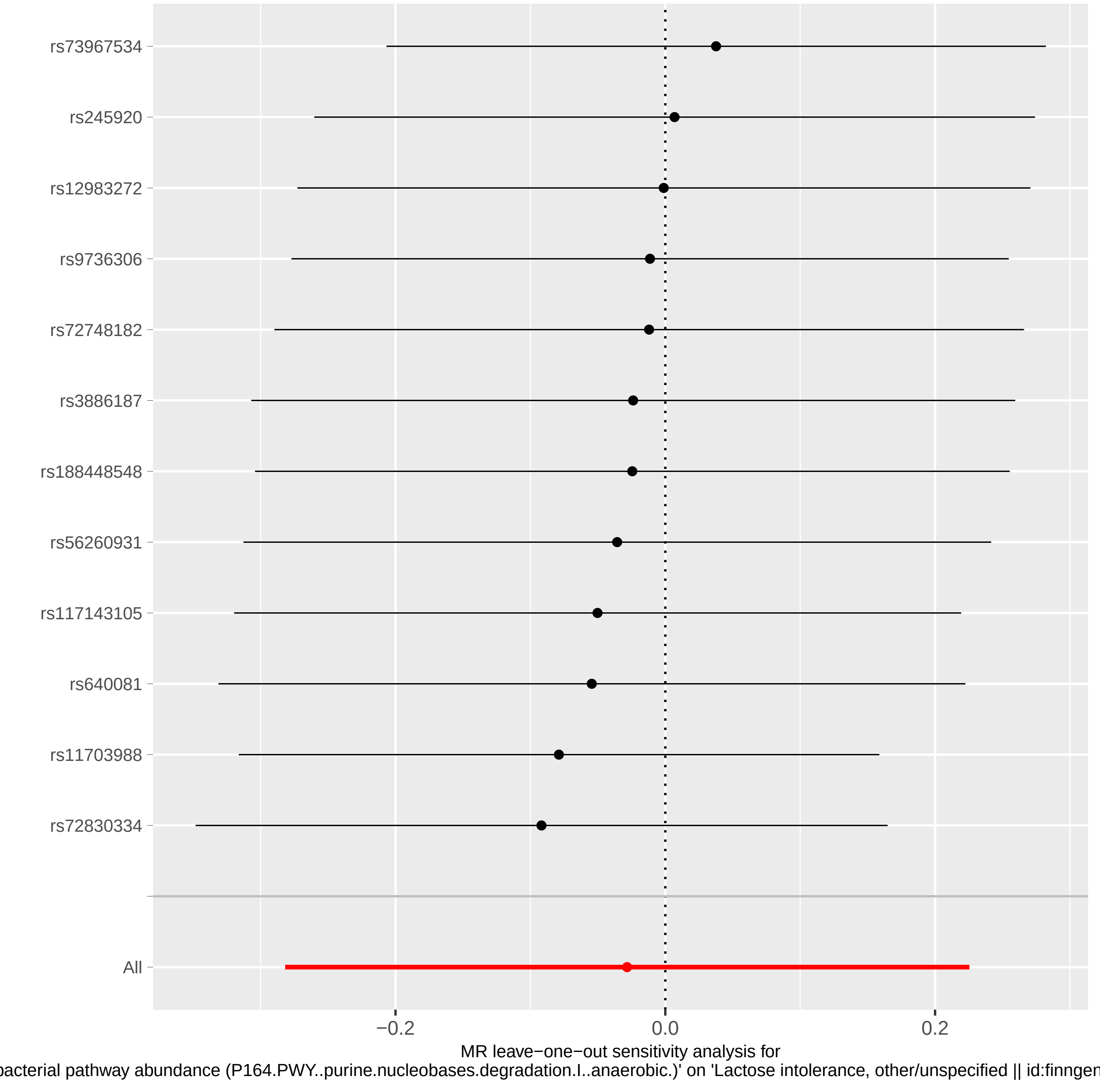
rs73967534
rs245920
rs12983272
rs9736306
rs72748182
rs3886187
rs188448548
rs56260931
rs117143105
rs640081
rs11703988
rs72830334
All
−0.2
0.0
0.2
MR leave−one−out sensitivity analysis for
bacterial pathway abundance (P164.PWY..purine.nucleobases.degradation.I..anaerobic.)' on 'Lactose intolerance, other/unspecified || id:finngen
